# Two rare intra-articsular knee disorders with overlapping symptoms: separate case reports of tenosynovial giant cell tumour and lipoma arborescens and literature review

**DOI:** 10.1186/s12891-026-09563-w

**Published:** 2026-02-10

**Authors:** Heqiang Chen, Xu Yang, Sihai Liu

**Affiliations:** 1 Department of Sports Medicine, Wuhan Fourth Hospital, Wuhan, 430033 China; 2 Berlin Institute of Health, Julius Wolff Institute for Biomechanics and Musculoskeletal Regeneration, Charité – Universitätsmedizin Berlin, Augustenburger Platz 1, Berlin, 13353 Germany

**Keywords:** Tenosynovial giant cell tumour, Lipoma arborescens, Magnetic resonance imaging, Arthroscopic synovectomy

## Abstract

**Background:**

Tenosynovial giant cell tumour (TGCT) and lipoma arborescens (LA) are both rare intra-articular lesions of the knee. Despite their distinct pathological nature, they often present with similar clinical manifestations such as joint swelling, pain, and limited motion, which makes differential diagnosis challenging.

**Case presentation:**

We report two patients who presented with progressive knee swelling and discomfort and were admitted within the same clinical session, offering a unique opportunity for direct comparison. Magnetic resonance imaging (MRI) revealed intra-articular soft-tissue masses in both cases. Both patients underwent arthroscopic synovectomy, with histopathological examination confirming the diagnosis of TGCT in one case and revealing the characteristic villous proliferation of adipose tissue within the synovium diagnostic of LA in the other. Both patients underwent surgical resection and experienced significant improvement in symptoms during follow-up.

**Conclusion:**

Although uncommon, TGCT and LA should be considered in the differential diagnosis of unexplained knee joint swelling or effusion. MRI provides valuable clues, but definitive diagnosis relies on histopathological confirmation. Awareness of these rare conditions may help avoid misdiagnosis and guide appropriate management.

## Introduction

Tenosynovial giant cell tumour (TGCT) is a rare mesenchymal neoplasm arising from the synovium of joints, bursae, and tendon sheaths. It encompasses two forms: a localized type (also historically termed giant cell tumour of tendon sheath) and a diffuse type (previously known as pigmented villonodular synovitis, PVNS) [1]. It is characterized by recurrent genomic alterations involving the colony-stimulating factor 1 (CSF1) gene [2]. According to the 2020 World Health Organization classification of soft tissue and bone tumours, TGCT is a locally aggressive lesion with minimal metastatic potential [3]. Although sarcomatous transformation with distant spread is exceedingly rare, the disease can significantly affect quality of life due to pain, swelling, and joint dysfunction. TGCT most commonly affects young adults, and its management remains challenging due to variability in treatment strategies and limited access to effective systemic therapies [4].

Lipoma arborescens (LA), also known as diffuse articular lipomatosis, is another rare intra-articular lesion characterized by subsynovial villous proliferation of mature adipose tissue, most frequently involving the suprapatellar pouch of the knee joint [5]. The term “arborescens” refers to its tree-like appearance, reflecting the frond-like morphology of the fatty villi. Unlike TGCT, LA is generally considered a benign reactive process associated with chronic irritation or degenerative joint disease rather than a neoplastic entity [6]. Clinically, LA presents as long-standing painless joint swelling, often accompanied by intermittent effusion and, later, pain [7]. While the knee is the most affected site, bilateral and polyarticular involvement has been reported in up to 20% of cases [8].

Although TGCT and LA are distinct entities, both can present with similar clinical features, making accurate preoperative diagnosis challenging. Imaging, particularly magnetic resonance imaging (MRI), plays a key role in differentiating these lesions, while histopathological examination remains the definitive diagnostic method. Herein, we report two rare cases of intra-articular lesions of the knee—one TGCT and one LA—who were admitted on the same day, to highlight their clinical presentation, imaging characteristics, and histopathological features, and to discuss their differential diagnosis and management strategies.

Herein, we report two rare cases of intra-articular knee lesions — 1 TGCT and 1 LA — both presenting on the same day to highlight their clinical presentation, imaging characteristics, and histopathological features, and to discuss their differential diagnosis and management strategies. The side-by-side presentation of these two distinct intra-articular pathologies within a narrow timeframe provides a unique clinical opportunity for comparative analysis of their overlapping yet distinct features.

## Case presentation

### Case 1: Tenosynovial giant cell tumour (TGCT)

A 15-year-old female presented with a 4-month history of progressive swelling and discomfort in the right knee. The symptoms were associated with a mild reduced range of motion, without a history of trauma or systemic illness. Physical examination revealed a joint effusion, evidenced by a positive patellar ballottement sign. There was no palpable warmth over the knee, and no periarticular tenderness was elicited on palpation. Passive and active flexion were mildly limited, with full extension maintained.

**Fig. 1:**
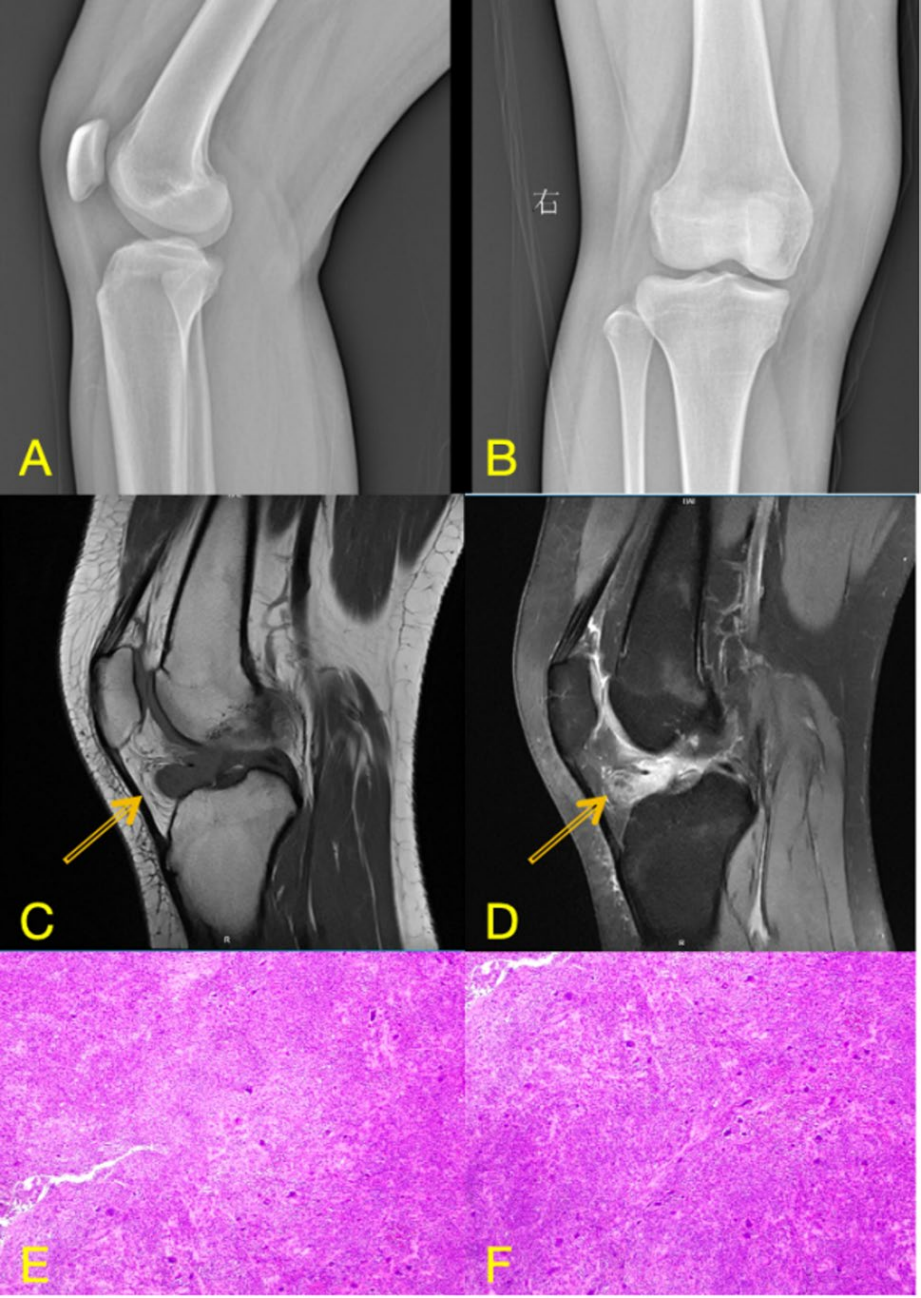
**A**, **B**: Preoperative knee radiographs in lateral (**A**) and anteroposterior (**B**) views; **C**, **D**: Corresponding MRI sequences: sagittal T2-weighted fat-suppressed image (**C**) and axial T1-weighted image (**D**); **E**, **F**: postoperative histopathological images

Preoperative anteroposterior and lateral radiographs of the right knee were unremarkable, showing no evidence of degenerative changes, calcifications, or osseous abnormalities. MRI of the right knee revealed an irregular focus of abnormal signal within the joint. The lesion (2.0 × 1.4 × 1.5 cm) demonstrated slightly high signal intensity on T2-weighted fat-suppressed images (T2WI-FS) and slightly low signal intensity on T1-weighted images (shown in Fig. 1). Its internal architecture appeared heterogeneous, featuring scattered punctate and linear hypointense foci, which are characteristic of hemosiderin deposition due to recurrent microhemorrhages within the lesion. Ill-defined margins were noted along the adjacent tibia, accompanied by localized cortical erosion and depression. The presence of hemosiderin is a key diagnostic clue for TGCT and accounts for the susceptibility artifacts commonly seen on gradient-echo sequences. These imaging findings, consistent with a solitary intra-articular mass exhibiting hemosiderin deposition and local bone involvement, are highly suggestive of a localized form of TGCT.

**Fig. 2:**
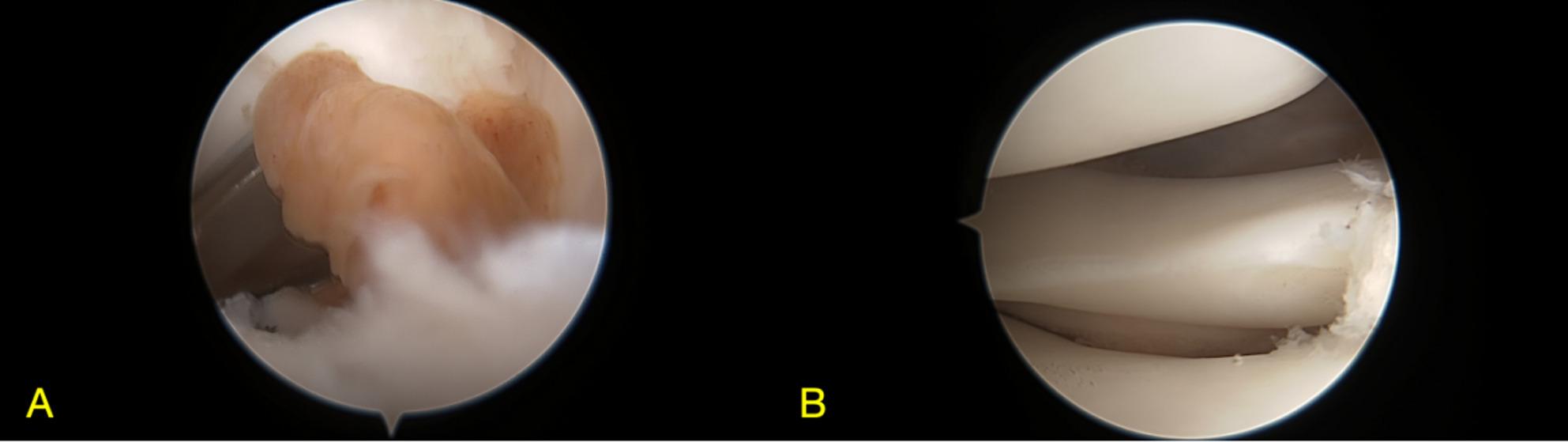
Arthroscopic images of the knee joint before (**A**) and after (**B**) excision of the localized nodular lesion

The patient underwent arthroscopic excision of the mass under spinal anesthesia. She was positioned supine with the affected right knee flexed at 90 degrees. The procedure was performed without the use of a tourniquet. Standard anterolateral and anteromedial portals were established. A solitary, brown, nodular lesion was discovered during surgery and completely resected. Histopathological examination confirmed a localized TGCT. The tumour tissue was composed predominantly of sheets of mononuclear cells with round to oval nuclei and eosinophilic cytoplasm. Scattered osteoclast-like multinucleated giant cells and focal collections of foam cells were observed. Fibrosis, hemosiderin deposition, and lymphocytic infiltration were noted in the interstitial tissue. Microscopically, a patchy distribution of tumour cells was observed (Fig. 2C, D). Immunohistochemically, the mononuclear and giant cells were positive for CD68 and CD163, with strong cytoplasmic expression. The lesional cells also showed diffuse positivity for Vimentin, while being negative for Desmin. The Ki-67 proliferation index was approximately 20% (shown in Fig. 2). Postoperative recovery was uneventful, and at 5-month follow-up, the patient was asymptomatic and showed no signs of recurrence.

### Case 2: Lipoma arborescens (LA)

A 25-year-old male presented with a 1-month history of progressive painless swelling of the right knee, associated with joint effusion and discomfort. There was no history of systemic disease or inflammatory arthropathy. However, a history of a minor right knee sprain prior to the onset of swelling was reported by this patient. On examination, suprapatellar fullness with mild restriction of movement. The knee demonstrated mild restriction of terminal flexion. A positive patellar tap sign confirmed a significant joint effusion. The skin temperature over the affected knee was slightly elevated compared to the contralateral side. Mild diffuse tenderness was present around the periarticular region, but no localized point tenderness was elicited.

**Fig. 3:**
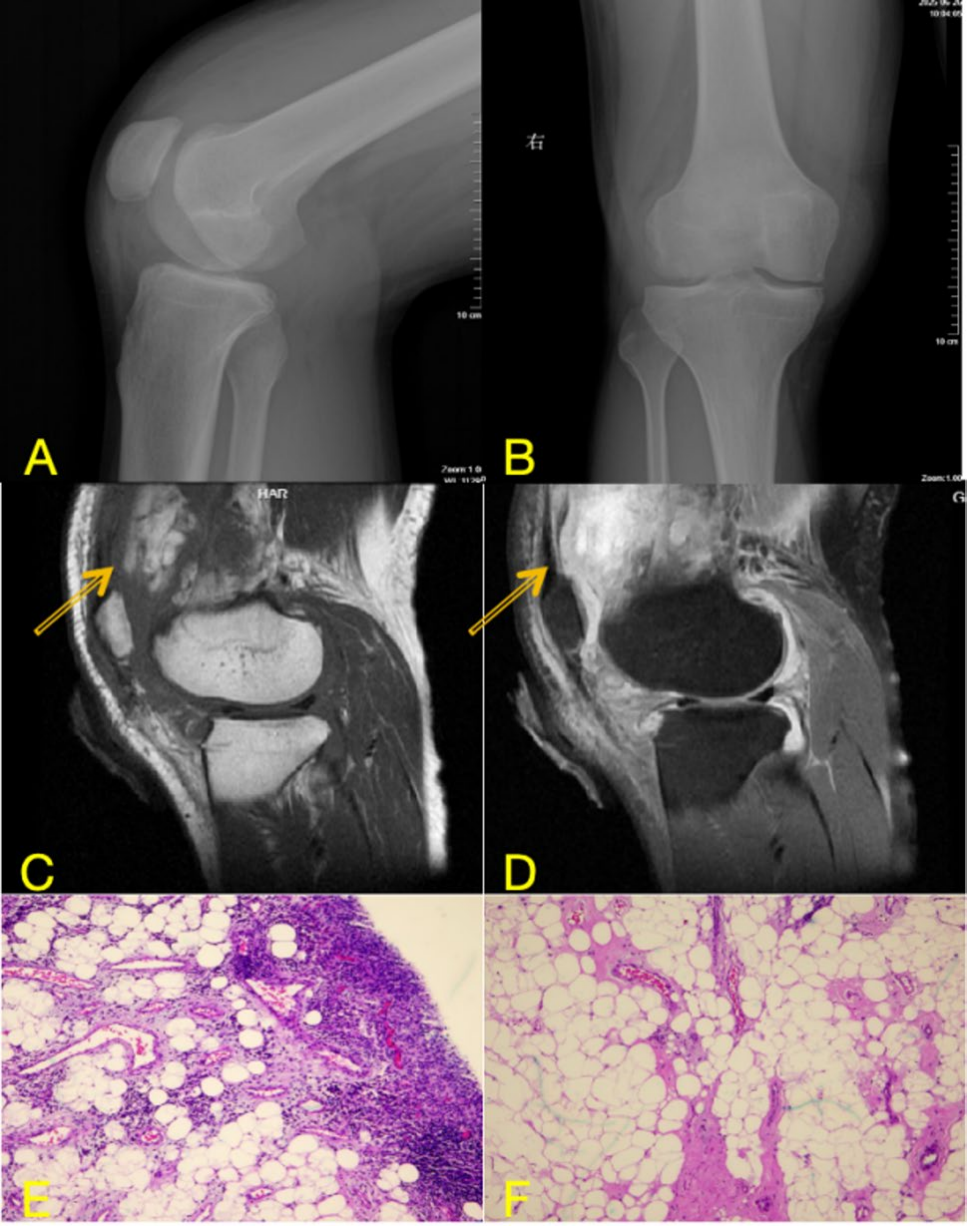
**A**, **B**: Preoperative knee radiographs in lateral (**A**) and anteroposterior (**B**) views; **C**, **D**: Corresponding MRI sequences: sagittal T2-weighted fat-suppressed image (**C**) and axial T1-weighted image (**D**); **E**, **F** postoperative histopathological images

Imaging demonstrated significant knee joint space narrowing, osteophyte formation at the tibial spine and patella, and adjacent soft-tissue swelling. The presence of significant joint space narrowing and osteophyte formation on radiographs indicates established osteoarthritis, which is incongruent with a purely acute one-month history. This suggests the possibility of a pre-existing, possibly subclinical, degenerative joint condition. MRI of the right knee demonstrated diffuse synovial thickening with arborizing, villous, and nodular morphological features throughout the joint, most prominently within the suprapatellar pouch. The lesions exhibit high signal intensity on T1-weighted images and signal loss on T2WI-FS sequences, consistent with fat-containing tissue (shown in Fig. 3). Associated findings include a joint effusion and soft tissue swelling surrounding the knee.

**Fig. 4:**
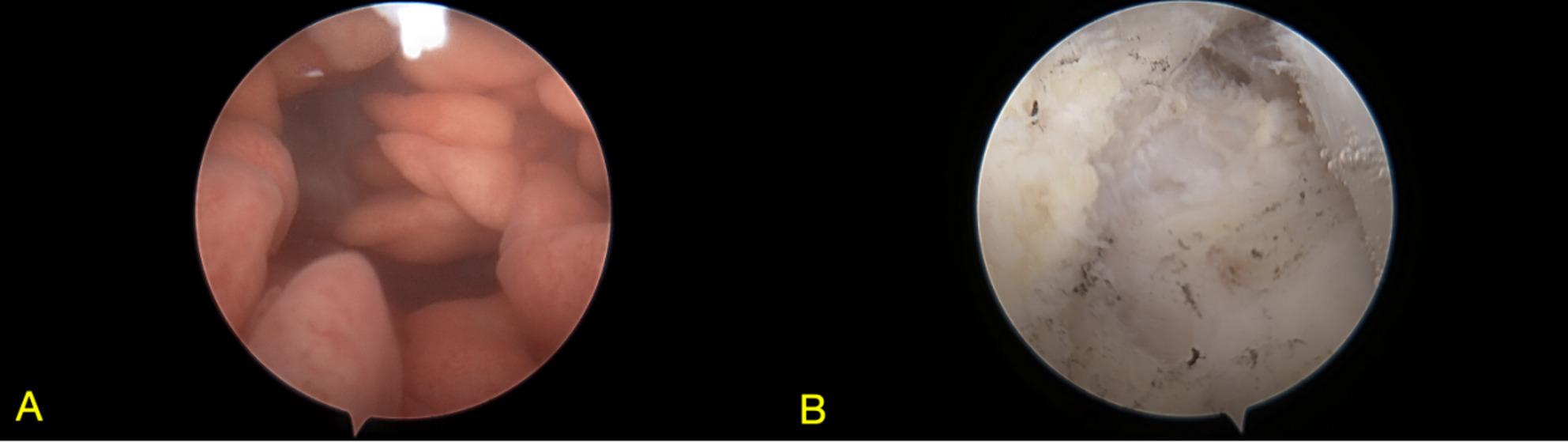
Arthroscopic view of the knee joint before (**A**) and after (**B**) synovectomy showing villous and arborizing fatty projections

The patient underwent arthroscopic synovectomy under spinal anesthesia in the supine position with the right knee flexed at 90 degrees, without a tourniquet. Access was achieved via standard anterolateral and anteromedial portals, supplemented by two posteromedial portals for optimal visualization. Intraoperatively, diffuse pale-yellow fatty tissue was observed within the joint, with the mass exhibiting lobulated and arborizing (tree-like) projections arising from the synovium. Histological analysis confirmed the diagnosis of LA. Microscopically, the synovium exhibited a characteristic lobulated and villous architectural pattern, with surface hyperplasia of the synovial lining cells. The subsynovial connective tissue was largely replaced by abundant, well-differentiated mature adipocytes arranged in distinct lobules. Prominent features included numerous dilated and congested capillaries within the fibrovascular cores of the villi, accompanied by a mild to moderate chronic inflammatory infiltrate composed predominantly of lymphocytes and plasma cells in the interstitial tissue. (shown in Fig. 4). The postoperative course was uneventful, and at 5-month follow-up, the patient remained asymptomatic with no evidence of recurrence.

## Discussion

This report leverages the side-by-side presentation of TGCT and LA within a single clinical setting to provide a focused comparative analysis that underscores key diagnostic challenges: despite sharing common non-specific symptoms such as knee swelling and discomfort, they are fundamentally different in pathogenesis, imaging appearance, and management [9–11]. By contrasting these two entities, we aim to enhance clinical awareness, facilitate accurate preoperative differentiation—primarily through MRI—and stress the indispensable role of histopathological confirmation. The following sections will systematically compare and contrast their epidemiology, pathogenesis, clinical and imaging features, and therapeutic approaches.

### Epidemiology and pathogenesis

TGCT is an uncommon proliferative disorder of synovial tissue that occurs in two distinct forms: a more common localized type and a less common diffuse type (the latter historically and synonymously referred to as PVNS). TGCT predominantly affects young adults, with a slight female predominance [12]. It arises from the synovium of joints and, less frequently, from tendon sheaths or bursae. Approximately 90% of TGCT cases present as the localized form, which appears as a solitary, well-circumscribed intra-articular or extra-articular mass [13]. The diffuse form (or PVNS) is found in a single joint compartment or the entire synovium, usually of large joints such as the knee, followed by the hip, shoulder, and ankle. This variant is more aggressive, often exceeding 5 cm, and is associated with a higher risk of recurrence and joint destruction [14].

The pathogenesis of TGCT is complex and involves both inflammatory and neoplastic components. Histologically, TGCT demonstrates a proliferative process within the synovium, characterized by stromal cell hyperplasia, mononuclear cell infiltration, and multinucleated giant cells of osteoclastic lineage [15, 16]. Hemosiderin deposition, resulting from recurrent intra-articular bleeding, is a hallmark feature and correlates with the low-signal intensity seen on MRI sequences [11]. While its etiology remains uncertain, TGCT shares certain features with rheumatoid arthritis-related inflammation, yet exhibits clonal cytogenetic abnormalities and, in rare cases, sarcomatous transformation with metastatic potential [17, 18]. This dual nature—part inflammatory, part neoplastic—has significant implications for therapeutic strategies, which draw upon approaches used in both fields.

LA is a rare benign synovial lesion characterized by villous lipomatous proliferation of subsynovial adipose tissue. It most commonly affects large joints, particularly the knee, although involvement of the hip, shoulder, ankle, wrist, and even tendon sheaths or bursae has been reported [19–21]. LA typically presents in adults between the fourth and sixth decades of life but has been described in both adolescents and elderly patients, with no apparent sex predilection [22, 23]. Most cases are monoarticular, but bilateral or polyarticular involvement can occur, especially in secondary forms [7, 8].

The exact etiology of LA remains unclear, but it is generally considered a benign reactive process associated with chronic irritation, trauma, or degenerative joint disease, rather than a true neoplasm [18]. In secondary forms, such as in our case where a history of minor knee sprain was reported, it may arise from chronic synovitis [24] or conversely, induce secondary inflammatory synovitis [25]. Primary LA, less common, occurs without an underlying joint disorder. Histologically, it is characterized by villous synovial hyperplasia with subsynovial replacement by mature adipocytes, without atypia [26]. Unlike liposarcoma, LA does not exhibit MDM2 gene amplification, which can be confirmed by fluorescence in situ hybridization (FISH) analysis [27]. While its pathogenesis may involve mesenchymal dysregulation, definitive molecular mechanisms are not yet fully defined.

### Clinical features

Both TGCT and LA can manifest as knee swelling and discomfort, which often complicates the initial clinical diagnosis. TGCT commonly affects young adults and may present with pain, stiffness, mechanical symptoms such as locking, and occasionally hemarthrosis [28]. In contrast, LA typically affects older adults and presents as a long-standing, painless swelling with intermittent joint effusion [22]. Both patients we reported presented with painless, progressive knee swelling and varying degrees of restricted knee movement. However, the disease duration was relatively short in our cases—four months for the TGCT patient and one month for the LA patient—likely attributable to early medical consultation and prompt diagnosis, a scenario that is less commonly described in the literature (Table 1).

**Table 1:** Key differentiating features between tenosynovial giant cell tumour (TGCT) and lipoma arborescens (LA)

Feature	Tenosynovial Giant Cell Tumour (TGCT)	Lipoma Arborescens (LA)
Typical Age	Young adults (20–40 years)	Older adults (40–60 years); also reported in adolescents and elderly
Clinical Onset	Progressive pain, swelling, stiffness, possible mechanical symptoms (locking)	Long-standing, often painless swelling; intermittent effusion
Common MRI Signs	Hypointense foci on T1 & T2 (due to hemosiderin); bone erosions may be present	Signal identical to fat: hyperintense on T1, signal loss on fat-suppressed sequences; frond-like morphology
Histopathology	Proliferation of mononuclear cells, multinucleated giant cells, foam cells; hemosiderin deposition	Villous synovial hyperplasia with subsynovial replacement by mature adipocytes; no atypia or lipoblasts
Nature	Locally aggressive neoplasm (clonal cytogenetic alterations)	Benign reactive process (non-neoplastic)
Common Treatment	Complete surgical excision (arthroscopic/open); CSF1R inhibitors for advanced cases	Arthroscopic or open synovectomy; often managed conservatively if asymptomatic
Recurrence Risk	Moderate (4–15% for localized form; higher for diffuse)	Very low; excellent prognosis after complete resection

The presentation of a 25-year-old male with radiographic evidence of established osteoarthritis (joint space narrowing, osteophytes) alongside LA is unusual. This discrepancy between the reported short symptomatic duration and chronic structural changes highlights a critical diagnostic challenge. It strongly suggests that the LA in this case is likely secondary to an underlying chronic joint disorder, such as post-traumatic or idiopathic early-onset osteoarthritis, rather than a primary process. The ‘acute’ one-month symptoms likely represent an exacerbation of chronic synovitis or increased effusion, bringing the condition to clinical attention. This scenario reinforces the argument for preoperative biopsy in young patients with atypical or mixed imaging findings, to differentiate true LA from other causes of fatty synovial proliferation and to clarify the primary versus secondary nature of the disease, which has implications for long-term management.

### Imaging and histopathological differentiation

Although TGCT and LA share overlapping clinical presentations, their distinct pathological underpinnings are clearly reflected in imaging and histopathological findings, which are pivotal for accurate diagnosis.

On MRI, which is the imaging modality of choice for both entities, characteristic differences are evident [29]. TGCT, as seen in our first case, typically presents as a nodular or diffuse synovial proliferation. Its MRI appearance is primarily influenced by hemosiderin deposition, manifesting as punctate or linear hypointensities on both T1- and T2-weighted sequences. In our Case 1, the lesion demonstrated atypically slightly high signal intensity on T2-weighted fat-suppressed images, which could reflect a mixture of hemosiderin with components such as subacute hemorrhage, cystic change, or edema. This finding underscores that atypical imaging features can occur in TGCT, thereby increasing diagnostic uncertainty and highlighting the importance of considering preoperative biopsy in ambiguous cases [30]. In contrast, LA demonstrates a unique, frond-like, arborizing synovial proliferation with signal intensity identical to that of subcutaneous fat, being hyperintense on T1-weighted images and exhibiting signal loss on fat-suppressed sequences [31]. The absence of significant hemosiderin-related susceptibility artifacts and the presence of this characteristic fat signal are key features that distinguish LA from TGCT and other synovial pathologies.

Definitive diagnosis, however, relies on histopathological examination, which reveals the fundamental difference in their nature. TGCT is characterized by a proliferation of mononuclear stromal cells, scattered osteoclast-like multinucleated giant cells, foam cells, and abundant hemosiderin deposition within the synovial tissue. This histologic profile supports its classification as a benign but locally aggressive neoplastic process [17, 32, 33]. Conversely, LA histology reveals villous synovial hyperplasia, characterized by the substitution of subsynovial connective tissue with mature adipocytes. The absence of atypia, mitotic figures, or lipoblasts confirms its benign, reactive nature [7, 10]. Thus, while MRI provides strong diagnostic clues, histopathology remains the gold standard, conclusively differentiating the neoplastic proliferation of TGCT from the lipomatous hyperplasia of LA.

### Differential diagnosis

The differential diagnosis for both lesions include other synovial proliferative disorders such as PVNS — the diffuse form of TGCT — synovial chondromatosis, synovial hemangioma, rheumatoid arthritis, gout, and liposarcoma [32–34]. MRI findings combined with clinical history help narrow the differential, but histological analysis remains essential to confirm the diagnosis and exclude malignancy.

### Treatment and prognosis

The primary treatment for localized TGCT is complete surgical excision, most commonly via arthroscopic or mini-open approach, with recurrence rates ranging from 4% to 15% depending on completeness of resection. Adjuvant radiotherapy or systemic therapy is generally reserved for diffuse or recurrent cases, as indicated in recent consensus guidelines [35]. Advances in understanding TGCT pathogenesis have led to targeted systemic therapies, particularly inhibitors of the CSF1 receptor (CSF1R) [2]. More recently, vimseltinib (DCC-3014), a highly selective CSF1R switch-control kinase inhibitor, has also shown promising efficacy and an acceptable safety profile trials, further validating this targeted approach [36, 37]. It is indicated for patients with symptomatic TGCT causing severe morbidity or functional limitations and not amenable to surgical resection. This approval represents a significant milestone in TGCT management and offers a therapeutic option for unresectable or recurrent cases [38].

While MRI is essential for differential diagnosis, its accuracy is not infallible. A recent TGCT study reported a 29% false-positive rate despite characteristic imaging findings, underscoring the need for preoperative biopsy in uncertain cases. In our report, proceeding directly to arthroscopic excision was based on high pretest probability and classical imaging features. However, in retrospect—particularly given the atypical T2 signal in Case 1 and unexpected OA changes in Case 2—a more cautious approach with preoperative biopsy would have provided definitive histologic confirmation prior to surgery, aligning better with contemporary best practices for atypical presentations.

Like many rare disorders, the management of LA lacks standardized evidence-based guidelines. Due to its benign behavior, asymptomatic cases may not necessitate immediate surgical intervention [39]. Nevertheless, the natural history of LA remains unclear in the absence of long-term follow-up studies. Surgical excision and synovectomy—whether performed via open or arthroscopic techniques—have been commonly employed, with reports indicating favorable short-term functional outcomes and no recurrence. Current systematic reviews support one-stage open or arthroscopic procedures as the treatment of choice, since they enable addressing both LA and concomitant intra-articular abnormalities—such as rotator cuff or labral tears [24, 40]. In secondary LA cases, managing underlying degenerative or inflammatory joint disease is crucial to minimize recurrence risk [26, 34]. With early diagnosis and appropriate treatment, both primary and secondary forms of LA have an excellent prognosis, as evidenced by our cases that remained recurrence-free throughout follow-up.

### Clinical implications

The similarity in clinical presentation of TGCT and LA underscores the importance of considering both entities in patients presenting with unexplained knee swelling or effusion. MRI remains an indispensable diagnostic tool, yet histological confirmation is mandatory before finalizing treatment decisions. Early recognition and management can prevent disease progression, reduce recurrence risk, and improve functional outcomes.

### Limitations

This study has several limitations. Although the follow-up period for both cases has been extended to five months, during which both patients remained asymptomatic with no evidence of recurrence on clinical assessment, this duration remains insufficient to fully evaluate the long-term recurrence risk, particularly for TGCT. We continue to monitor both patients prospectively and plan further clinical and MRI evaluations at the 12-month postoperative mark to better assess oncological outcomes. Second, the decision to proceed directly to arthroscopic excision without preoperative biopsy, while clinically justified in these specific contexts given the high pre-test probability and classical imaging features (for LA), may not represent the standard approach in all centers, particularly for lesions with more ambiguous characteristics. Finally, the novelty of this report is largely contingent upon the rare coincidence of both pathologies presenting on the same day, which may limit its generalizability.

## Conclusion

TGCT and LA, though rare and distinct in nature, can present with similar clinical manifestations, particularly knee swelling and effusion. MRI serves as the cornerstone of preoperative evaluation, while histopathology remains essential for definitive diagnosis. Early surgical intervention yields excellent outcomes for both entities. Furthermore, molecular advances such as CSF1R inhibitors have expanded therapeutic options for unresectable or recurrent TGCT. Timely recognition and management are critical to preserving joint function and preventing recurrence. The juxtaposition of these two cases within a condensed timeframe reinforces the importance of considering both entities in differential diagnosis and demonstrates the value of direct comparative analysis in clinical learning.

## Data Availability

The data supporting the findings of this case are available from the corresponding author upon reasonable request.

## Abbreviations

TGCT: Tenosynovial giant cell tumour
LA: Lipoma arborescens
MRI: Magnetic resonance imaging
PVNS: Pigmented villonodular synovitis
CSF-1: Colony-stimulating factor 1
T2WI-FS: T2-weighted fat-suppressed images
FISH: Fluorescence in situ hybridization
CSF1R: Colony-stimulating factor 1 receptor
